# An end to coercion: rights and decision-making in mental health care

**DOI:** 10.2471/BLT.19.234906

**Published:** 2019-10-17

**Authors:** Kanna Sugiura, Faraaz Mahomed, Shekhar Saxena, Vikram Patel

**Affiliations:** a Department of Mental Health, Graduate School of Medicine, The University of Tokyo, Tokyo, Japan.; b Harvard Law School Project on Disability, Harvard University, Cambridge, United States of America (USA).; c Department of Global Health and Population, Harvard T. H. Chan School of Public Health, Boston, USA.; d Department of Global Health and Social Medicine, Harvard Medical School, 641 Huntington Avenue, Boston, Massachusetts, MA02115, USA.

## Abstract

The *United Nations Convention on the Rights of Persons with Disabilities* requires a paradigm shift from a medical model of disability to a social model that emphasizes overcoming the barriers to equality created by attitudes, laws, government policies and the social, economic and political environment. The approach adopted by the social model recognizes that people with psychosocial disabilities have the same right to take decisions and make choices as other people, particularly regarding treatment, and have the right to equal recognition before the law. Consequently, direct or supported decision-making should be the norm and there should be no substitute decision-making. Although recent mental health laws in some countries have attempted to realize a rights-based approach to decision-making by reducing coercion, implementing the *Convention on the Rights of Persons with Disabilities* can be challenging because it requires continuous refinement and the development of alternatives to coercion. This article reviews the impact historical trends and current mental health frameworks have had on the rights affected by the practice of involuntary treatment and describes some legal and organizational initiatives that have been undertaken to promote noncoercive services and supported decision-making. The evidence and examples presented could provide the foundation for developing a context-appropriate approach to implementing supported decision-making in mental health care.

## Résumé

La Convention des Nations Unies relative aux droits des personnes handicapées nécessite un changement radical pour passer d'un modèle médical du handicap à un modèle social mettant l'accent sur la levée des obstacles en matière d'égalité, créés par des attitudes, des lois, des politiques gouvernementales ainsi que l'environnement social, économique et politique. L'approche adoptée par le modèle social reconnaît que les personnes présentant un handicap mental ont le même droit de prendre des décisions et de faire des choix que les autres, notamment en matière de traitement, et ont droit à une reconnaissance égale devant la loi. Par conséquent, la prise de décision directe ou accompagnée doit être la norme et il ne doit exister aucune prise de décision substitutive. Bien que certains pays aient récemment cherché, à travers des lois sur la santé mentale, à instaurer une approche fondée sur les droits en matière de prise de décision en réduisant la coercition, la mise en œuvre de la Convention relative aux droits des personnes handicapées peut poser problème, car elle exige une amélioration continue et l'élaboration de solutions autres que la coercition. Cet article examine l'impact que les tendances historiques et les cadres actuels en matière de santé mentale ont eu sur les droits lésés par la pratique du traitement involontaire et décrit plusieurs initiatives législatives et organisationnelles qui ont été prises pour promouvoir les services non coercitifs et la prise de décision accompagnée. Les données et les exemples présentés peuvent servir de fondement à l'élaboration d'une approche adaptée au contexte pour mettre en place la prise de décision accompagnée en matière de soins de santé mentale.

## Resumen

La Convención de las Naciones Unidas sobre los Derechos de las Personas con Discapacidad requiere un cambio de paradigma, de un modelo médico de discapacidad a un modelo social que haga hincapié en la superación de las barreras a la igualdad creadas por las actitudes, las leyes, las políticas gubernamentales y el entorno social, económico y político. El enfoque adoptado por el modelo social reconoce que las personas con discapacidad psicosocial tienen el mismo derecho a tomar decisiones y a elegir como cualquier otra persona, especialmente en lo que se refiere al tratamiento, y tienen derecho a un reconocimiento igualitario ante la ley. Por lo tanto, la toma de decisiones directa o apoyada debería ser la norma y no debería haber un responsable sustituto de la toma de decisiones. Aunque las recientes leyes sobre salud mental de algunos países han tratado de aplicar un enfoque basado en los derechos para la adopción de decisiones mediante la reducción de la coerción, la implementación de la Convención sobre los Derechos de las Personas con Discapacidad puede ser un reto, ya que requiere un continuo perfeccionamiento y el desarrollo de alternativas a la coerción. Este artículo evalúa el impacto que las tendencias históricas y los marcos actuales de salud mental han tenido sobre los derechos afectados por la práctica del tratamiento involuntario y describe algunas iniciativas legales y organizativas que se han emprendido para promover servicios no coercitivos y apoyar la toma de decisiones. La evidencia y los ejemplos presentados podrían servir de base para desarrollar un enfoque apropiado al contexto para la implementación de la toma de decisiones apoyada en la atención de la salud mental.

## ملخص

تتطلب اتفاقية الأمم المتحدة لحقوق الأشخاص ذوي الإعاقة، تغييراً نوعياً من نموذج طبي للإعاقة إلى نموذج اجتماعي يركز على التغلب على العقبات أمام المساواة، وينشأ عن المواقف والقوانين والسياسات الحكومية والبيئة الاجتماعية والاقتصادية والسياسية. يدرك الأسلوب الذي ينتهجه النموذج الاجتماعي أن للأشخاص الذين يعانون من الإعاقات النفسية الاجتماعية، ذات الحق في اتخاذ القرارات وتحديد الاختيارات مثلهم مثل الآخرين، وخاصة فيما يتعلق بالعلاج، ولديهم لحق في التقدير المكافئ أمام القانون. وبالتبعية، ينبغي أن يكون صنع القرار المباشر أو المدعوم هو الأساس، ويجب ألا يكون هناك صنع لقارات بديلة. على الرغم من أن قوانين الصحة العقلية الحديثة في بعض البلدان قد حاولت تنفيذ أسلوب قائم على الحقوق لصنع القرارات عن طريق الحد من الإكراه، إلا أن تنفيذ اتفاقية حقوق الأشخاص ذوي الإعاقة يمكن أن يمثل تحديًا لأنه يتطلب تحسيناً مستمرًا وتطوير بدائل للإكراه . يستعرض هذا المقال التأثير الذي تركته الاتجاهات التاريخية وأطر الصحة العقلية الحالية على الحقوق المعنية بممارسة العلاج غير الطوعي، ويصف بعض المبادرات القانونية والتنظيمية التي تم الاضطلاع بها للارتقاء بالخدمات غير القسرية ودعم عملية صنع القرار. إن الأدلة والأمثلة الواردة يمكنها أن تمثل حجر الأساس لتطوير أسلوب مناسب للسياق لدعم عملية صنع القرار في مجال الرعاية الصحية العقلية.

## 摘要

联合国《残疾人权利公约》要求从残疾医学模式向强调平等的社会模式转变，在这种模式下需要克服因态度、法律、政府政策以及社会、经济和政治环境造成的不平等。社会模式所采用的方法认为患有社会心理障碍的人与他人一样有权作决策和选择，特别是在治疗方面，并有权在法律面前获得平等认可。因此，直接或受支持的决策应该成为规范，不应由他人替代作出决策。尽管最近一些国家的心理健康法试图通过减少胁迫来实现基于权利的决策方法，但执行《残疾人权利公约》可能会十分棘手，因为这需要不断完善和发展替代强制的办法。本文回顾了历史趋势和当前的心理健康框架对受非自愿治疗的权利所产生的影响，并描述了为促进非强制服务和支持决策所采取的一些法律和组织措施。所提供的证据和范例可以制定一种适用于具体情况的方法，此方法可为实施心理健康护理的支持性决策奠定基础。

## Резюме

Конвенция Организации Объединенных Наций о правах инвалидов требует коренного перехода с медицинской на социальную модель понимания инвалидности, которая делает акцент на преодолении барьеров на пути к равенству, создаваемых менталитетом, законами, политикой правительств, а также социальной, экономической и политической обстановкой. Социальная модель понимания инвалидности признает за людьми с психосоциальной инвалидностью те же права на принятие решений и наличие выбора, что и за другими людьми, в частности в том, что касается лечения, а также признает их право на равенство перед законом. Следовательно, нормой должно быть принятие решения человеком, которого оно непосредственно касается, или принятие решения с поддержкой, а не принятие решения представителем. Несмотря на то что недавно принятые законы о психиатрической помощи в некоторых странах предпринимают попытку реализовать подход к принятию решений на основе соблюдения прав человека и уменьшить уровень принуждения, осуществление Конвенции о правах инвалидов может быть непростым делом, так как оно требует постоянного уточнения и разработки альтернатив принуждению. В статье рассматривается влияние, оказываемое историческими тенденциями и существующими системами психиатрической помощи на права человека, затронутые практикой принудительного лечения, и описываются некоторые юридические и организационные инициативы, которые предпринимаются для пропаганды услуг непринудительного характера и принятия решений с поддержкой. Представленные в статье свидетельства и примеры могут стать основой для разработки соответствующего контексту подхода к принятию решений с поддержкой в практике психиатрической помощи.

## Introduction

In December 2018, an independent review of the 1983 Mental Health Act of the United Kingdom of Great Britain and Northern Ireland concluded that reforms were needed to reduce coercion in mental health care and to support mental health service users in making their own decisions about treatment. The review stated that, “allowing everyone to make the decisions that affect their life and accept the consequences of those decisions is a key aspect of respecting the unique value and character of each human person.”^1^ Similarly, in 2019, the Council of Europe’s Commissioner for Human Rights, Dunja Mijatović, noted that,

“Historically, rejection and isolation has been our default response to persons with psychosocial disabilities. This ingrained fear is still very strong and is fuelling the prejudice that they [persons with psychosocial disabilities] are automatically a danger to themselves and to society, against all available statistical evidence to the contrary.”^2^

In making these comments, Mijatović recognized the relationship between coercive care, isolation from the community and the stigmatization of people living with psychosocial disabilities (i.e. disabilities arising from the interaction between a person with a mental health condition and their environment). Stigmatization remains a challenge and may ultimately lead to the violation of numerous rights, such as the right to live freely in the community, and the right to make decisions about treatment or support. The underlying belief is that people with psychosocial disabilities lack the intellectual capacity to make decisions for themselves, which can engender a destructive cycle of marginalization and abuse.^3^ The harmful effects of coercion have led commentators, such as the United Nations’ Special Rapporteur on torture and other cruel, inhuman and degrading treatment or punishment, Juan Mendez, and Mijatović to propose that there should be no coercion under any circumstances.^2^^,^^4^

Full realization of the human rights of people with psychosocial disabilities is a general principle of the World Health Organization’s (WHO’s) *Comprehensive mental health action plan 2013–2020*.^5^ With the issue becoming a central concern for policy-makers and practitioners alike, there is a need to consider how this general principle can be operationalized in the context of decision-making. The aims of this paper are to highlight the human rights implications of involuntary mental health treatment and admission, to examine the consequences of this practice, and to explore the operationalization of a rights-oriented approach to decision-making and legal capacity in a range of scenarios. In addition, given that mental health conditions are distributed across a spectrum, and that the paradigm espoused by the *United Nations Convention on the Rights of Persons with Disabilities* should be incorporated into care and support regimes throughout that spectrum, the paper also considers the legal capacity challenges faced by people with acute conditions because their situation has given rise to the most complex debates among both practitioners and scholars.^6^^–^^8^

## Rights affected by involuntary treatment

Historically, mental health systems have been too reliant on coercion and have tended to deny that people with mental health conditions have the capacity to decide whether to accept or refuse treatment.^9^ Moreover, a key characteristic of mental health laws around the world has been substitute decision-making, whereby the decision of a clinician or another official can legally supersede the preference of an individual if that individual is deemed to be mentally incapacitated. Although these provisions are intended to protect people with mental health conditions from harm or from causing harm to themselves or others, scholars and activists have documented cases in which substitute decision-making has led to abuses, ranging from the use of psychiatric methods to suppress political dissent to the sexual and physical abuse of mental health service users in the custody of psychiatrists.^10^^,^^11^ Along with over-reliance on coercion, involuntary institutionalization has often been used to deal with people with serious mental health conditions despite a lack of clear clinical evidence supporting the practice.^12^

The Convention on the Rights of Persons with Disabilities, which was introduced in 2007, has been viewed as a radical step forward in the support and care of people with disabilities. Article 12 of the Convention states that they have a right to equal recognition before the law and General Comment 1 on Article 12 (adopted in 2014) states that all persons possess decision-making capacity, which means that substitute decision-making is inconsistent with the right to equal recognition before the law. Instead, the Convention and General Comment 1 mandate supported decision-making, whereby the necessary accommodations are made (and support provided) to ensure that individuals can express their own will and preferences. In rare instances in which individuals may be unable to do so, practitioners and other officials should make every effort to arrive at the most accurate interpretation of the individual’s will and preferences. The Convention is one of the most widely ratified treaties in history, to date there are 177 state parties. In 2017, it was reported that at least 32 countries had either undertaken, or were in the process of implementing, reforms to their mental health frameworks to incorporate the paradigm advanced by the Convention.^13^ Signature and ratification of the Convention mandate each state to ensure its provisions are fully applied in domestic laws, policies and practices.

Involuntary treatment or admission conflicts with the principle of autonomy, a central guiding principle of the Convention. Moreover, the acceptability and quality of any form of coercive mental health care has been questioned. There is evidence that the effects of coercive treatment lead to substantial trauma,^14^ that its putative benefits cannot be sustainably maintained,^15^ and that fear of coercion can actually deter help-seeking behaviour.^16^ In contrast, detractors of the Convention’s approach have argued that universal application of the Convention’s provisions may, in itself, violate the right to health because people who might need treatment in an emergency or who might be at risk of harming themselves or others may not receive it, this would contravene their right to treatment and risk further impairment.^17^ While the debate continues, there is increasing evidence to support the efficacy of noncoercive models of care that align closely with the principles of dignity and autonomy and that do not nullify the right to treatment. These models include community-based interventions and practices that emphasize the will, and preferences of mental health service users, as described below. The right to health is, therefore, better served by these more acceptable practices.

The right to equality is also affected by coercive practices because they deny that everyone has an equal capacity to make decisions about their own well-being. Similarly, the right to inclusion in the community is violated by coercive practices that can result in institutionalization or in another form of marginalization. Community inclusion is not only a fundamental right, but as research suggests, it is also an important component of well-being because it contributes to both the prevention and treatment of serious mental health conditions.^18^ Although inclusion in the community may be challenging when people experience acute distress or exhibit a propensity to harm themselves or others, there should always be a presumption against restricting their right to inclusion arbitrarily or unreasonably.^19^ Failure to uphold this presumption merely exacerbates the stigmatization and marginalization of people with psychosocial disabilities and can, as a result, present a considerable barrier to accessing services.^20^

The right to be protected from cruel, inhumane and degrading treatment has also been invoked by people concerned about the harm that can be caused by involuntary mental health treatment. In 2013, the United Nations’ Special Rapporteur on torture and other cruel, inhuman and degrading treatment or punishment called on states to “impose an absolute ban on all forced and non-consensual medical interventions…including the non-consensual administration of psychosurgery, electroshock and mind-altering drugs [and] the use of restraint.”^4^ Similarly, the Special Rapporteur’s 2017 report on the right to physical and mental health noted that, despite its questionable clinical effectiveness and the rights violations that may occur, involuntary mental health treatment continues to be a common practice.^21^ The report calls on states to, “radically reduce medical coercion and facilitate the move towards an end to all forced psychiatric treatment and confinement.”

## A rights-based approach to decision-making

Avoiding coercion and realizing supported decision-making in mental health services involves paying systematic attention to all relevant rights and incorporating them into national laws, policies and programmes. Adopting a context-specific approach to achieving the goals of the Convention is important, because differences in resources might necessitate different approaches, and because local social, cultural and political factors may influence implementation. The supported decision-making paradigm of the Convention can be realized by implementing legislative measures, by increasing the participation of mental health service users in treatment and policy-making, and by providing community-based care and support.

### Legislative measures

According to WHO’s Mental Health Atlas 2017, 111 countries (i.e. 57% of all WHO Member States) reported having a stand-alone law for mental health and 66 reported having updated that law in the previous 5 years. The Atlas states that 39% of all Member States (76 countries) have a mental health law that is “partially or fully in line with international human rights instruments.” In addition, 139 countries (i.e. 72% of WHO Member States) reported having a stand-alone policy or plan for mental health and 120 (i.e. 62% of Member States) reported having updated that policy or plan in the previous 5 years. The Atlas states that 48% (i.e. 94 Member States) have a mental health policy or plan that is “partially or fully in line with international human rights instruments.”^22^ Many countries have developed legislative and policy tools aimed at operationalizing a rights-based approach to decision-making and legal capacity. We searched for country examples by contacting key informants and following up on examples described in WHO’s QualityRights initiative, supplemented by our own knowledge.^23^ Box 1 describes some notable examples we identified of the legal approaches to meeting obligations incumbent upon state parties to the Convention. These examples demonstrate that efforts are being made to incorporate supported decision-making into legislation in a range of contexts around the world. However, they also illustrate that this area of law-making presents challenges and that there continues to be a reliance on some form of coercion despite considerable efforts to avoid it.

Box 1 **Examples of mental health legislation, worldwide, 2011–2017**

**Canada (British Columbia)**

The Mental Health Act of British Columbia (2011) enables mental health service users to issue advance directives that explicitly state their will and preferences in the event of a mental health crisis. However, the Act contains provisions that allow physicians to determine whether involuntary treatment or hospital admission is warranted, albeit with safeguards, such as periodic reviews.^24^

**China**

China passed its first mental health law in 2012. This law aims to guarantee the legal rights and interests of persons with mental health conditions. However, it also contains a provision for guardianship and requires guardians to safeguard the legal rights and interests of persons with mental health conditions.^25^

**Costa Rica**

In 2016, Costa Rican mental health law created the legal figure of a “guarantor for equality before the law,” whose role is to ensure the personal autonomy of an individual with a mental health condition. The law also fully abolished guardianship.^26^

**India**

The Indian Mental Health Act of 2017 requires informed consent for the administration of mental health services and medication. It also allows for substitute decision-making when an individual is said to have “ceased” to possess the capacity to make decisions themselves. The Act provides for advance directives and a Mental Health Review Board was established to enable mental health service users to contest their admission to hospital or report any violation by, or deficiency in, mental health services.^27^

**Peru**

Although decision-making regimes in Peru are covered by the civil code, the General Law on Persons with Disabilities (2012) and subsequent amendments affirm that legal capacity is universal. This outcome resulted from the close involvement in the drafting process of people with psychosocial disabilities and disabled people’s organizations. Nonetheless, the Law still allows involuntary treatment in emergencies and for people with addiction.^28^

**United Kingdom (Northern Ireland)**

The 2016 Mental Capacity Act (Northern Ireland) is an example of “fusion” legislation. Fusion legislation treats people with mental and physical health conditions in the same way when an intervention is proposed and focuses instead on impairments in decision-making capacity. Consequently, fusion legislation reduces the stigmatization of mental health conditions and discourages the overuse of substitute decision-making for people with health limitations. However, a person’s best interests may still be determined by a substitute.^29^

### Participation of mental health service users

Participation is another key principle of the Convention. Involuntary mental health treatment, by its nature, constitutes a denial of this right, as do structural barriers to participation in policy-making. Engaging with mental health service users themselves, both on individual treatment choices and on policy-making is therefore needed. Those efforts can involve mental health advisory committees, monitoring bodies and advocacy structures. Input from mental health service users can also be solicited directly through different platforms, such as social media.^30^ Moreover, policy-makers, researchers and clinicians may themselves be mental health service users. Recruiting people with experience of a serious mental health condition into organizations that address concerns arising out of involuntary mental health treatment can provide a powerful impetus for change and can lead to better clinical outcomes.^31^

By carrying out a scoping exercise and engaging with key informants, we identified important measures that can be taken to foster participation and inclusive decision-making, such as co-production and patient-centred outcomes research. Again, these measures can be applied in a multitude of contexts and to mental health conditions of any severity. Co-production refers to a relationship in which power and the responsibility to plan and deliver support are shared between professionals and mental health service users. Co-production ensures that people with mental health conditions are consulted, included and participate in decision-making from the start to the end of any project that affects them.^32^ In patient-centred outcomes research and user-led research, mental health service users are engaged in research, not simply as subjects but as partners who help determine what should be studied and how. This approach should shift the focus of research onto the topics, questions and outcomes that are most important to patients and their caregivers. Many disabled people's organizations are involved in identifying the needs of mental health service users, evaluating services and advocating for change and public awareness. In fact, the inclusion of Article 12 in the Convention resulted from advocacy by the World Network of Users and Survivors of Psychiatry.^33^

### Community-based care and support

Community-based care and support are explicitly intended to avoid the need for hospital admission. In addition, this approach can also incorporate supported decision-making that respects the rights of people with psychosocial disabilities and has been shown to have the added benefit of reducing stigmatization.^34^ Moreover, there is evidence that community-based care and support can be applied in different ways in countries as varied as Finland, India and Mexico,^35^^–^^37^ which demonstrates that a lack of resources should not be considered an impediment to realizing the Convention’s vision. This approach has been found to be viable for people with acute episodes of mental health conditions as well as for less severe cases.^38^ Box 2 describes the varied ways supported decision-making has been implemented around the world, which we identified by carrying out a scoping exercise and engaging with key informants. Box 2 also highlights the diversity of the methods used to realize the rights of mental health service users, many of which could be replicated elsewhere. Although most of these methods have been empirically validated, others require additional research to establish their efficacy.

Box 2 **Types of community-based, supported decision-making for mental health service users**

**Peer support^39^^,^^40^**

Supported decision-making regimes that include peer support inherently advance the right to participation and to care and support in a community of peers. These regimes should, therefore, be incorporated into mental health and psychosocial support services.

**Circle of support^41^^,^^42^**

A circle of support is the group of family members, friends, peer supporters and supportive workers who provide support and friendship to a mental health service user. These individuals can suggest ideas, provide support with planning or help implement plans by engaging with mental health service users in a way that enables them to express their will and preferences in a safe and supportive environment.

**Open dialogue^43^**

An open dialogue involves the mental health service user, family members, clinicians and other relevant people who meet soon after a crisis. In the dialogue, the emphasis is on responding to the needs of the whole person rather than on eradicating symptoms. Uncertainty is embraced to encourage open conversation and avoid reaching a premature conclusion. Open dialogue is effective in reducing the need for hospitalization and medication and in returning the mental health service user to a previous level of functioning.^43^

**Circle of care^44^^,^^45^**

The circle of care comprises members of the health-care team providing ongoing care for the mental health service user, it may include doctors, nurses, pharmacists, psychologists, social workers and other health-care providers. This format encourages a patient-centred approach, supports the mental health service user and facilitates the collection, use, disclosure and handling of personal health information for providing direct health care or for decision-making.

**Personal ombudsman^46^**

A personal ombudsman is a skilled individual who helps his or her client with a wide range of issues, such as family matters, housing, accessing services and employment. The personal ombudsman should be able to argue effectively for the client’s rights with authorities or in court. The client must establish a relationship, and start a dialogue, with the personal ombudsman before he or she is engaged.

**Crisis plan^47^^,^^48^**

A crisis plan is a document that outlines the actions that should be taken to aid recovery when a person is unwell. It can be developed by the person, with or without the help of others, and is an effective and enforceable legal document. The crisis plan can state what the person wants others to do. Implemented together with a post-crisis plan, it can identify and reduce risks to the person.

**Crisis card^49^**

A crisis card is a small card that a person can carry and which contains information about what to do and whom to contact in the event of a crisis. The card can be presented to anyone, including friends, health-care professionals, police officers and bystanders.

**Crisis care centre or house^50^**

A crisis care centre is a facility to which an individual can go in a crisis to stabilize, detox, find respite or identify the services they need. These centres provide an alternative to inpatient psychiatric care and help the individual engage with the support system.

## Conclusion

Adopting a rights-based approach to decision-making in mental health care primarily involves: (i) aligning mental health laws more closely with the Convention on the Rights of Persons with Disabilities; (ii) fostering the participation of mental health service users in policy and decision-making; and (iii) establishing community-based strategies for supported decision-making. These practices have been adopted in a range of economic and cultural contexts, and have been applied to mental health conditions of all degrees of severity. They have the potential to lessen the stigma faced by people with psychosocial disabilities, to reduce discrimination against them, and to ensure their will and preferences are paramount in all decisions that affect them. Although some aspects of substitute decision-making are still common, these innovative practices can provide a strong foundation for transforming mental health services. However, these practices need to be replicated and research is required to evaluate their impact, and identify ways of entrenching their adoption in practice. In addressing coercion in mental health, the first step should always be to examine the specific context in which the issues and concerns arise; any assessment should identify: (i) the people most affected; (ii) the problems that result from coercion; (iii) the people or organizations that have an obligation to do something about the situation; (iv) the capacities and resources available to take action to rectify the situation; and (v) the challenges that might develop in seeking to address the problem. In keeping with a rights-based approach, it is paramount that the interventions applied should be readily available, accessible, acceptable and of a high quality. As we demonstrated above, this can be done in various contexts under a range of conditions. Ultimately, the principles we have outlined represent an opportunity to realize a rights-based approach to mental health care, one that should not be missed.
